# Unexpected presentation of allograft dysfunction triggered by page kidney phenomenon immediately after kidney transplantation: a case report

**DOI:** 10.1186/s12882-018-0860-2

**Published:** 2018-03-13

**Authors:** Shunta Hori, Mitsuru Tomizawa, Fumisato Maesaka, Takuya Owari, Yosuke Morizawa, Yasushi Nakai, Makito Miyake, Tatsuo Yoneda, Nobumichi Tanaka, Katsunori Yoshida, Kiyohide Fujimoto

**Affiliations:** Department of Urology, Nara Medical University, 840 Shijo-cho, Kashihara, Nara, 634-8522 Japan

**Keywords:** Renal transplantation, Page kidney, Allograft cyst

## Abstract

**Background:**

Page kidney phenomenon is caused by strong renal parenchymal compression and leads to renal hypoperfusion and microvascular ischemia, resulting in renal dysfunction and hypertension. Although the development of Page kidney phenomenon in allograft is rare, most of its cases are induced by allograft biopsy or trauma. We observed a case of Page kidney phenomenon that was induced by unusual causes immediately after kidney transplantation.

**Case presentation:**

A 66-year-old man, whose wife donated a kidney, underwent ABO-compatible living kidney transplantation. The allograft had three renal arteries that were trimmed and formed into one piece on the back table, and subsequently, it was anastomosed to the internal iliac artery. Intraoperative Doppler ultrasonography (US) revealed adequate blood flow of each renal artery. Urine output was also observed as soon as allograft blood flow was reperfused. After the surgery, the urine output decreased, and serum creatinine level increased to 6.0 mg/dL. Doppler US did not show evidence of acute rejection, ureteral obstruction, or anastomotic stenosis of the renal arteries. On postoperative day 7, surgical exploration was performed and revealed that the blood flow of each renal artery was adequate but subcapsular hematoma was detected at the upper pole of the allograft. Capsulotomy and hematoma evacuation were performed. Subsequently, urine output increased and serum creatinine level decreased up to 1.7 mg/dL. Allograft sample was obtained 1 h after the transplantation from the lower pole of the allograft. Although the cause of subcapsular bleeding was unclear in this case, a small cyst of the allograft, which might have ruptured during donor nephrectomy, was located in the middle of the hematoma, and oozing around the cyst was observed.

**Conclusions:**

Our case indicated that the small ruptured cyst of the allograft could be the cause of subcapsular hematoma and Page kidney phenomenon. Subcapsular hematoma caused by oozing over time could be difficult to diagnose using Doppler US, and thus, other imaging modalities, such as computed tomography, should be considered. Knowledge of the Page kidney phenomenon in the allograft can lead to early diagnosis and intervention, resulting in better outcomes for recipients with allograft dysfunction.

## Background

Page kidney phenomenon (PK) is caused by strong renal parenchymal compression, leading to renal hypoperfusion and microvascular ischemia. Reduction of renal blood flow causes renal dysfunction and activation of the renin–angiotensin–aldosterone axis, resulting in the development of hypertension [1, 2]. Although the occurrence of PK is rare in allograft, some recipients develop PK because of subcapsular hematomas after allograft biopsy, peritransplant lymphocele, or trauma. Doppler ultrasonography (US) is regarded as an important tool for the early diagnosis of PK and may reveal findings such as subcapsular hematomas and elevated resistive index (RI) [3–6]. In our case, these signs were undetected even with repeated examination by Doppler US. We observed an unexpected presentation of allograft dysfunction induced by PK immediately after renal transplantation.

## Case presentation

**Fig. 1:**
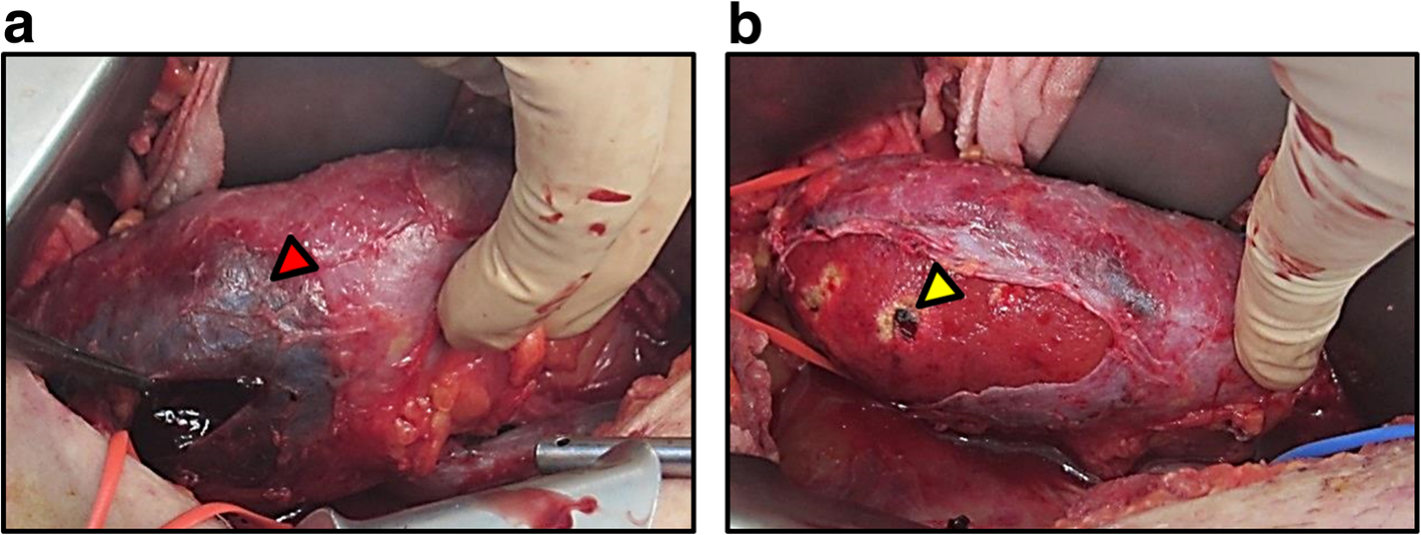
Representative images of allograft at the time of surgical exploration. Subcapsular hematoma was confirmed at the upper pole of the allograft. Capsulotomy was performed, and hematoma with clots was observed (red arrow) and subsequently evacuated (**a**). After decompression, the allograft parenchyma expanded and turned bright red (yellow arrow), indicating improvement of blood flow at the upper pole of allograft (**b**)

**Fig. 2:**
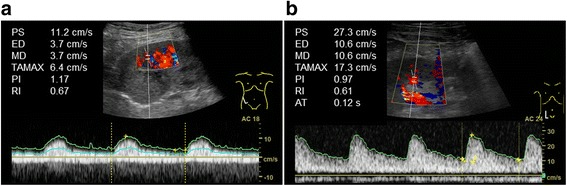
Representative images of allograft taken by Doppler ultrasonography. Before capsulotomy and evacuation, blood flow of the upper pole of the allograft was slightly poor, and diastolic flow decreased (end-diastolic velocity = 3.7 cm/s, resistive index = 0.67) (**a**). After decompression, allograft blood flow improved (end-diastolic velocity = 10.6 cm/s, resistive index = 0.61) (**b**)

**Fig. 3:**
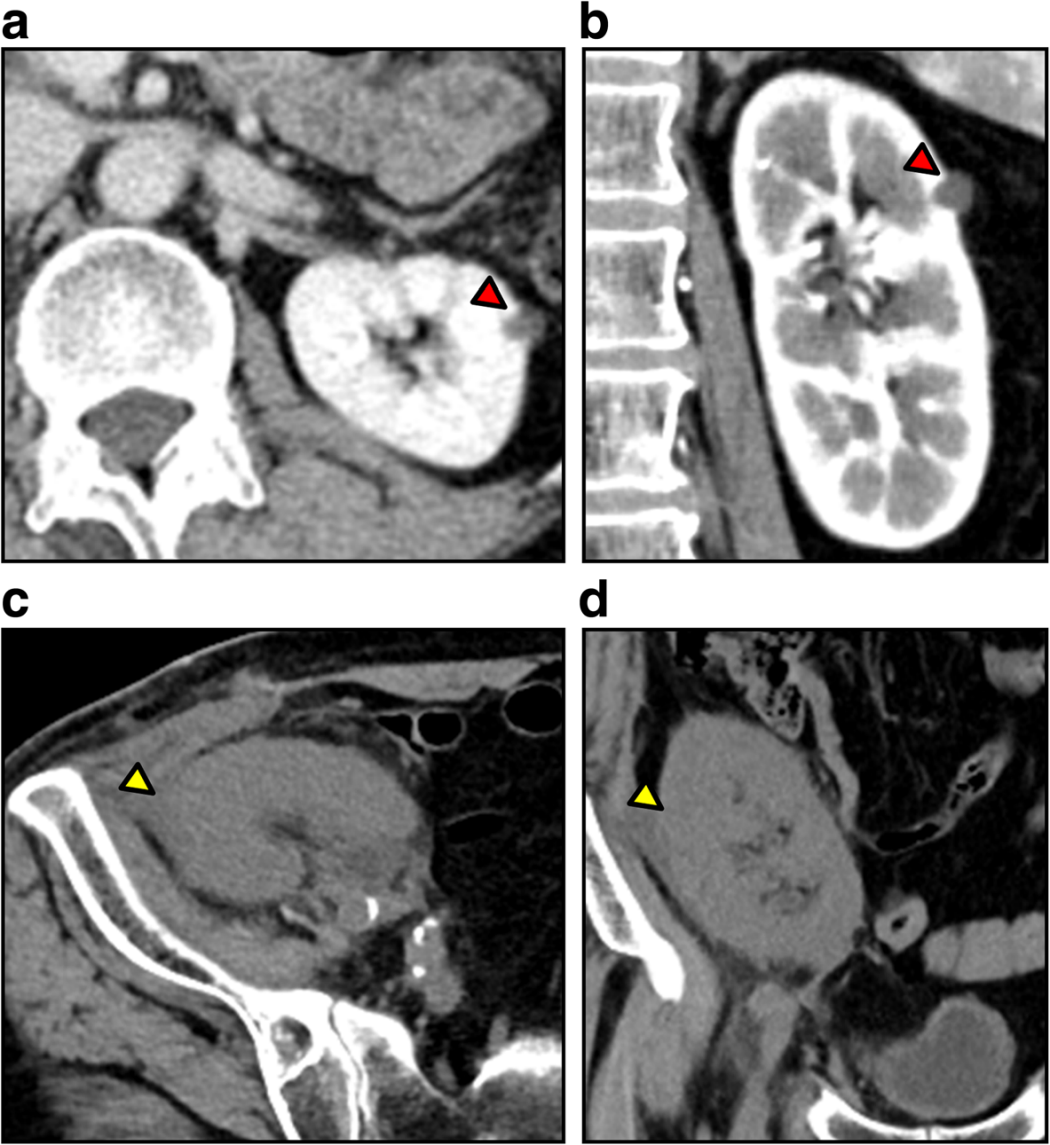
Representative images of allograft obtained by enhanced or unenhanced computed tomography. Preoperative enhanced computed tomography of allograft shows a small renal cyst at the middle to upper pole of the allograft (red arrow: (**a**); axial image, (**b**); coronal image). In the postoperative unenhanced image of the allograft, a small cyst detected before transplantation was unclear (yellow arrow: (**c**); axial image, (**d**); coronal image)

**Fig. 4:**
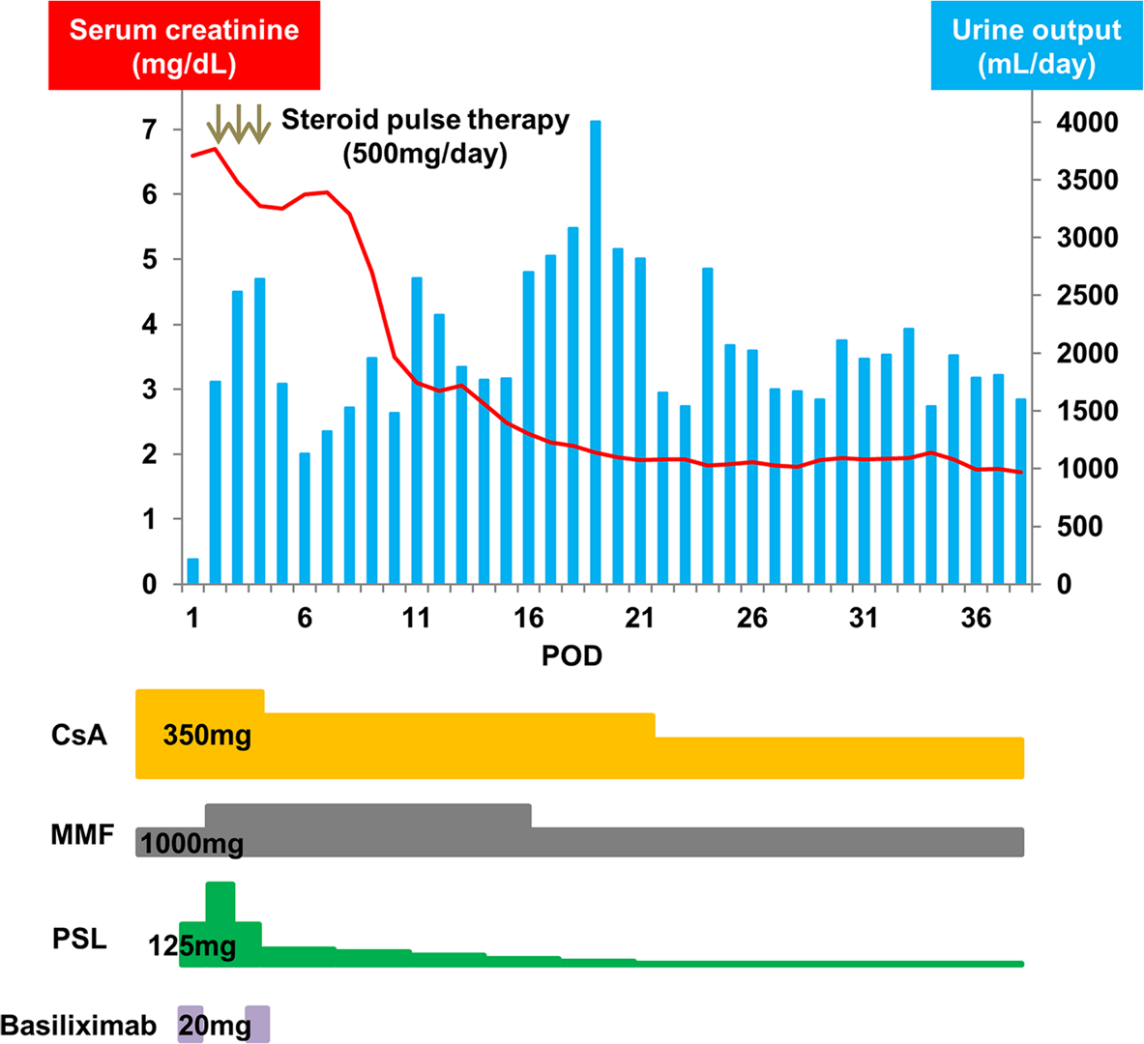
Clinical course of serum creatinine level and urine output, and the regimen of introduced immunosuppression. On postoperative day 6, serum creatinine level was 6.0 mg/dL, and urine output decreased. On postoperative day 7, surgical exploration was performed. After decompression of the allograft, serum creatinine level decreased to 1.7 mg/dL, and urine output increased. The immunosuppression regimen was as follows: cyclosporin, mycophenolate mofetil, prednisone, and basiliximab. At discharge, the doses of each immunosuppressive agent were as follows: 150 mg/day cyclosporin, 1000 mg/day mycophenolate mofetil, and 5 mg/day prednisone. On the day of transplantation and postoperative day 4, 20 mg/day basiliximab was administered. POD = postoperative day; CsA = cyclosporine; MMF = mycophenolate mofetil; PSL = prednisone

A 66-year-old man who underwent regular hemodialysis (4 h a day, 3 times a week) for 9 months for end-stage renal disease secondary to IgA nephropathy was hospitalized for living kidney transplantation. He underwent ABO-compatible living kidney transplantation. The kidney donor was his wife; the allograft had three renal arteries that were trimmed and formed into one on the back table, and subsequently, it was anastomosed to the internal iliac artery. Intraoperative Doppler US revealed that the blood flow of each renal artery was adequate, resulting in sufficient blood flow throughout the allograft. Urine output was also observed as soon as blood flow returned. Allograft biopsy an hour after allograft blood flow returned was performed at the lower pole of the allograft, and we confirmed no bleeding from the operative field, including the biopsy site. His introduced immunosuppression regimen included cyclosporin, mycophenolate mofetil, prednisone, and basiliximab. Although urine output was observed postoperatively, serum creatinine level did not decrease. Doppler US showed no evidence of acute rejection, ureteral obstruction, or anastomotic stenosis of renal arteries on postoperative day (POD) 2. Although rejection was considered and steroid pulse therapy was initiated, the allograft function did not improve. On the contrary, urine output decreased, and serum creatinine level increased to 6.0 mg/dL. On POD 6, Doppler US did not show evidence of rejection and anastomotic stenosis, but a region with less blood flow was observed at the upper pole of the allograft, as well as a slight decrease in diastolic flow. During this period, his blood pressure was 145/80 mmHg, and progression of anemia was not observed. On POD 7, surgical exploration was performed and revealed that the blood flow of each renal artery was sufficient, but subcapsular hematoma was detected at the upper pole of the allograft (Fig. 1a). Capsulotomy was performed, and hematoma with clots was evacuated. Ruptured cyst was observed in the middle of the hematoma, and slight oozing around the cyst was observed (Fig. 1b). Allograft decompression improved the blood flow at the upper pole of the allograft, and diastolic flow was also improved (Fig. 2). Retrospectively, preoperative enhanced computed tomography (CT) of the donor showed a small renal cyst at the upper pole of the allograft (Fig. 3a and b), and postoperative unenhanced CT of the recipient showed that the cyst was unclear (Fig. 3c and d). At the same time, allograft biopsy was performed, which did not show evidence of rejection and calcineurin inhibitor toxicity, but acute tubular injury was induced by allograft ischemia. After capsulotomy followed by evacuation, urine output increased, and serum creatinine level decreased to 1.7 mg/dL (Fig. 4).

## Discussion

The occurrence of PK is rare in allograft, and Cromie et al. first reported PK as pseudorejection in 1976 [7]. PK induces renal microvascular ischemia and causes allograft dysfunction. Acute PK can possibly restore allograft function with appropriate treatment, including capsulotomy and hematoma evacuation. Therefore, early recognition is very important. The most common reason for PK in the allograft is subcapsular hematoma due to allograft biopsy. Chung et al. reported that acute pain over the allograft, alteration in blood pressure control, and reduction of urine output were useful signs to diagnose early-stage PK, and early intervention, such as evacuation via capsulotomy and compression-free abdominal closure, led to good prognosis. They also stated that findings of subcapsular hematoma and elevated RI by Doppler US led to early diagnosis [6]. In this case, Doppler US, which was performed on every POD, did not show evidence of subcapsular hematoma and elevated RI, resulting in exploratory laparotomy. Decrease in diastolic flow is not sufficient to diagnose PK. Although CT scan can be performed conveniently and provide significant information, CT was not performed in this case because of the reliability of Doppler US. Therefore, we reaffirmed that examination from various angles using various modalities is very important.

Reportedly, PK for allograft is caused mainly by allograft biopsy and trauma. Risk factors for the development of PK for allograft are not well established [5, 8]. Takahashi et al. summarized previous reports, and the most common causes were iatrogenic, meaning allograft biopsy and three cases lost their allograft function [5]. Although the cause of subcapsular bleeding was unclear in this case, a small cyst of the allograft, which might rupture at donor nephrectomy, was located in the middle of hematoma, and oozing around the cyst was observed. Thus, we speculate that the time of oozing around the ruptured cyst was longer before subcapsular hematoma formed, resulting in renal parenchymal compression. Butt et al. reported a case with an unusual presentation of PK in allograft and suggested that even without any apparent traumatic cause or symptoms, such as allograft dysfunction and hypertension, PK should not be ruled out [9]. In our case, the presentation was also atypical and unexpected; thus, differentiating the cause of allograft dysfunction was difficult. Considering PK is important for accurate early diagnosis and early intervention when allograft dysfunction is observed. Furthermore, performing donor nephrectomy is important, and careful observation of the allograft is also required during the surgery.

Allograft dysfunction induced by PK could be improved by appropriate treatment before irreversible damage occurs [5]. In this case, allograft function improved, as evidenced by a serum creatinine level of 1.7 mg/dL after capsulotomy and hematoma evacuation. However, the decrease in serum creatinine level was not sufficient. Although we speculated that partial ischemic condition of up to 7 days resulted in the allograft function, leading to an irreversible damage, allograft biopsy showed acute tubular injury induced by allograft ischemia. Allograft biopsy also showed no evidence of rejection and calcineurin inhibitor toxicity, which often caused allograft dysfunction during the perioperative period. In addition, ischemia–reperfusion injury might have an adverse effect on the allograft function. Although the use of vasopressor to maintain urine output in the perioperative period might be one of the reasons for delayed diagnosis because of masking of hypertension, the allograft function might improve if early intervention with capsulotomy and hematoma evacuation is possible.

In our experience, considering PK a cause of allograft dysfunction might lead to early and accurate diagnosis and prevention of allograft loss. Although Doppler US has a high diagnostic capability for PK, some cases cannot be diagnosed with Doppler US. Multiple imaging modalities should be used without hesitation if they are less harmful to the allograft.

## Conclusion

We reported an unexpected presentation of PK in the allograft secondary to oozing from a small ruptured cyst of the allograft. Considering PK could lead to early diagnosis and early intervention, resulting in the prevention of allograft loss.

## Abbreviations

CT: Computed tomography
PK: Page kidney phenomenon
POD: Postoperative day
RI: Resistive index
US: ultrasonography

## References

[1] Page IH (1939). The production of persistent arterial hypertension by cellophane perinephritis. JAMA.

[2] Engel WJ, Page IH (1955). Hypertension due to renal compression resulting from subcapsular hematoma. J Urol.

[3] Machida J, Kitani K, Inadome A, Wada Y, Kawabata K, Yoshida M, Ueda S (1996). Subcapsular hematoma and hypertension following percutaneous needle biopsy of a transplanted kidney. Int J Urol.

[4] Yussim A, Shmuely D, Levy J, Servadio C, Shapira Z (1988). Page kidney phenomenon in kidney allograft following peritransplant lymphocele. Urology.

[5] Takahashi K, Prashar R, Putchakayala KG, Kane WJ, Denny JE, Kim DY, Malinzak LE (2017). Allograft loss from acute page kidney secondary to trauma after kidney transplantation. World J Transplant.

[6] Chung J, Caumartin Y, Warren J, Luke PP (2008). Acute page kidney following renal allograft biopsy: a complication requiring early recognition and treatment. Am J Transplant.

[7] Cromie WJ, Jordan MH, Leapman SB (1976). Pseudorejection: the page kidney phenomenon in renal allografts. J Urol.

[8] Gainza FJ, Minguela I, Lopez-Vidaur I, Ruiz LM, Lampreabe I (1995). Evaluation of complications due to percutaneous renal biopsy in allografts and native kidneys with color-coded Doppler sonography. Clin Nephrol.

[9] Butt FK, Seawright AH, Kokko KE, Hawxby AM (2010). An unusual presentation of a page kidney 24 days after transplantation: case report. Transplant Proc.

